# Measles second dose vaccine uptake and its associated factors among children aged 24–35 months in Northwest Ethiopia, 2022

**DOI:** 10.1038/s41598-024-61048-9

**Published:** 2024-05-14

**Authors:** Worku Taffie, Habtamu Temesgen, Wassachew Ashebir, Habitamu Mekonen

**Affiliations:** 1 East Gojjam Zone, Shebel Berenta Woreda Health Office, Debre Markos, Ethiopia; 2 Department of Human Nutrition, College of Health Science, Debre Markos University, Debre Markos, Ethiopia; 3 Department of Public Health, College of Health Science, Debre Markos University, Debre Markos, Ethiopia

**Keywords:** Measles, Measles second dose vaccine, Children, Immunization, Vaccination, Immunology, Diseases, Health care

## Abstract

Measles is a major public health problem in under-five children, leading to lifelong complications. Therefore, the study aimed to assess the magnitude of measles second-dose vaccine uptake and its determinants among children aged 24–35 months in Northwest Ethiopia. A community-based cross-sectional study was conducted among 418 children aged 24–35 months in Northwest Ethiopia between January 2022 and February 2022. A simple random sampling technique was used to access study subjects. A binary logistic regression model was employed. An adjusted odd ratio with a 95% confidence interval (CI) and a p-value < 0.05 was used to declare significant predictors of measles second dose vaccine uptake. The magnitude of the measles second dose vaccine uptake among children aged 24–35 months was 41.39%. Postnatal care visits (AOR: 4.78, CI 1.49, 15.34), child vaccination status of other scheduled vaccines (AOR: 3.88, CI 2.23, 6.73), awareness of the measles second dose vaccine and its schedule (AOR: 8.924, CI 5.27, 15.09), and distance from the vaccination center (AOR: 0.21, CI 0.06, 0.77) were significantly associated with measles second dose vaccine uptake. The uptake of measles second dose vaccine in the study area was low. Therefore, health workers and other partners should initiate awareness creation programs for mothers/caretaker to improve the uptake of measles second dose vaccine.

## Introduction

Measles is one of the most highly contagious human pathogens; a single case of measles results in 12 to 18 secondary cases^1^. It is characterized by fever, maculopapular erythematous rash, cough, and conjunctivitis. Measles spreads through respiratory droplets when an infected person coughs or sneezes^2,3^. To track the high burden of measles disease, the World Health Organization (WHO) recommends coverage of ≥ 95% of two doses of measles vaccine (MCV)^4,5^. Although global second-dose measles vaccination coverage nearly quadrupled from 18 in 2000 to 71% in 2019^6^, measles is still a public health problem in under-five children, attributed to more than 140,000 global deaths^7^ and 15,000 to 60,000 cases of blindness annually globally^3^. More than 95% of measles deaths and morbidities occur in countries with low incomes and weak health systems^8^. These outbreaks have been attributed to insufficient vaccination rates and individuals with vaccine-hesitant attitudes^9^. Of 159,073 global measles cases reported in 2020, 77% were found in the African region^10^. Measles epidemics frequently strike various parts of Ethiopia, putting 650,000 children at risk^11^. From 2006 to 2016, there were 66,719 confirmed cases in Ethiopia, and measles incidence increased from 20 cases per million in 2006 to 49 per million in 2016, and of all cases, 28% were under-five children^12^. As per the WHO in Africa report of 2022, there were 2755 suspected cases of measles in Ethiopia, out of which 2156 cases were confirmed cases^13^. In the Amhara National Regional State, the area of interest, the analysis of 7296 samples reveals that 2412 (36.7%) were found to be measles positive^12^.

When the first dose of the measles vaccine is administered to children ages 9 to 12 months, the seroconversion rate is about 85%. This increases to 90–95% when children are vaccinated at 12 months, and it is even higher if the vaccine is administered at 15 months^14,15^. Increased routine measles vaccine coverage with two doses of measles vaccine (MCV2) and supplementary immunization activities have resulted in remarkable progress in reducing measles incidence and mortality^14^. Along with this, a strong, well-functioning immunization program that achieves high coverage of the measles second dose vaccine is considered the standard of care for high population immunity against ^16,17^.

Globally, due to the COVID-19 pandemic, about 1.32 million children under 1 year old did not receive the measles vaccin^18^. The Worldwide Measles Elimination Progress Report from 2000 to 2019 was alarming and drove actions towards the regional measles elimination targets by reaching all children with second doses of the measles-containing vaccine ^19^. In Ethiopia, a national measles-containing second-dose vaccine (MCV2) introduction was launched on February 11, 2019, to be administered at the age of 15–18 months^20^. Globally, according to a WHO report in 2020, 73% of under-five-year-old children received the second dose of the measles-containing vaccine, with significant regional variations, of which the lowest coverage was in the African region (25%)^21^. In Ethiopia, based on mini-EDHS 2019, only 9% of under-five-year-old children received MCV2, while measles-containing first-dose vaccine coverage is 59%^22^. Along with this, a seasonal pattern of occurrence of measles outbreaks has been observed over the years^23^. Sustaining physician awareness, high vaccination coverage, and elimination-standard surveillance are key strategies to eliminate the measles disease^24^. Several factors contribute to the low coverage of MCV2 in many sub-Saharan countries. Studies revealed that the uptake of the measles second dose vaccine was affected by the mother's age, education level, socio-economic status, marital status, religion, parity, perception, and knowledge level, the child’s gender, birth order, distance from the health facility, antenatal care, place of delivery, and utilisation of other vaccines (MCV1, pentavalent, oral polio vaccine (OPV), pneumococcal vaccine, and vitamin A supplementation)^25–29^.

Although several interventions have been tried by the country’s government and partners, the national measles-containing vaccination target remains far from being achieved^16^. This low coverage of measles second-dose vaccination creates a conducive environment for sustained measles outbreaks^30^. Measles immunization, particularly measles second-dose vaccine coverage, is a key indicator in the Sustainable Development Goals and Immunization Agenda 2030^31^. Based on the level of measles vaccination coverage and transmission rate, the timing of MCV1 and MCV2 administration may vary, and thus, every opportunity should be taken to vaccinate under-five-year-old children^16^. Therefore, the age range of 24–35 months is an appropriate age category to get complete information regarding MCV2 vaccination status, and children in the age range of 24–35 months have almost the same birth cohort as children less than 24 months. As a newly implemented immunization program, little is known about the status of measles-containing second-dose vaccine uptake, as well as its contributing factors. Thus, information regarding the level of measles second dose vaccination and its determinants is needed to suggest appropriate interventions that improve the uptake of measles second dose vaccine. Therefore, this study aimed to assess measles second dose vaccine uptake and its associated factors among children aged 24–35 months in Northwest Ethiopia.

## Methods

### Study setting, design and period

A community-based cross-sectional study was conducted in Shebel Berenta district in northwest Ethiopia between January 2022 and February 2022. Shebel Berenta is one of the 22 districts in Northwest Ethiopia, located 372 km from Bahir Dar, the capital city of Amhara National Regional State. It is bordered on the south by Dejen District, on the north by Enarj Enawuga, and on the east by the Abay River^32^. It is located at an elevation of 1800–2150 m above sea level. Approximately 72.3% of the district is desert (kola), with Woyinadega accounting for the rest (

**Figure 1:**
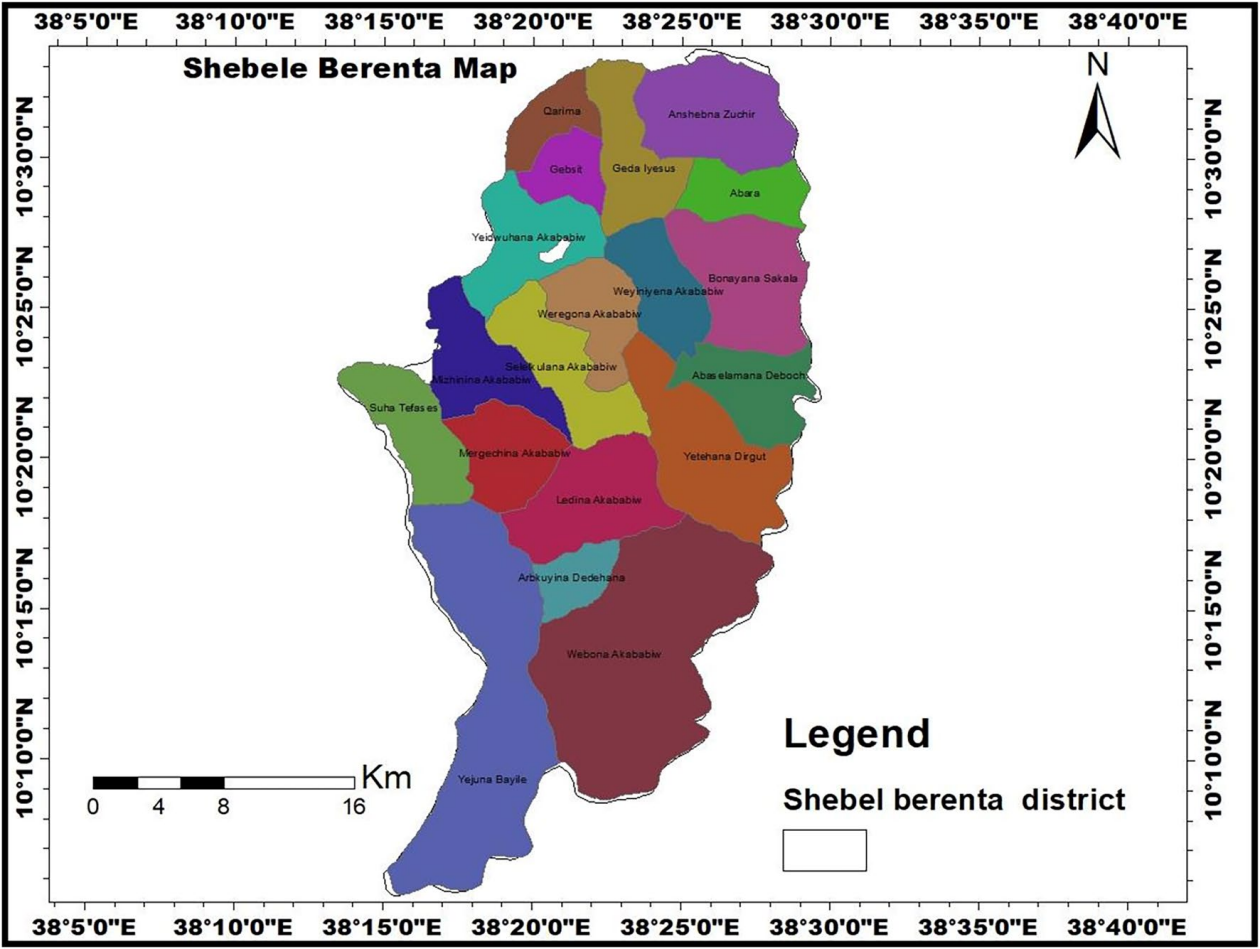
Map of the study area (Shebel Berenta district) (generated using GIS software version 10.5; URL: https://arcgis.software.informer.com/10.5/).

Figure 1)^33^, and the map of the study area of interest, Shebele Berenta was generated. Based on the 2021 population projection, the district has an estimated population of 136,948. Of those, 68,200 are males and 68,748 are females. There are nineteen kebels (15 rural and 4 urban kebeles) in the district. There are 6 health centers, 23 health posts, 1 primary hospital, 4 private clinics, and 3 drug stores that provide community health services^34^.

### Population, sample size and sampling techniques

The source population was all mothers or caregivers with children aged less than 60 months who have been residing in Shebel Berenta for at least six months. Whereas mothers/caretaker with children aged 24–35 months who lived in selected kebeles of Shebel Berenta district for at least six months and received the first dose of the measles vaccine were the study population. We exclude mothers/caretakers with children aged 24–35 months who did not receive the first dose of measles vaccination, were ill or unable to respond, and/or residents of less than six months in the study area.

The sample size was calculated using the single population proportion formula by considering the following assumptions of sample size determination in a cross-sectional study: 50% proportion of MCV2 uptake (since there are no previous studies in the study area of interest), a 95% level of confidence interval (CI), and a margin of error of 5%. Then, the sample size of the study was determined using the general formula:

$${\text{n}}=\frac{{({\text{Z}}1-\mathrm{\alpha }/2)}^{2}*pq}{{(d)}^{2}}=\frac{{(3.84)}^{2}*0.5*0.5}{{(0.05)}^{2}}=385$$

where n = minimum required sample size, p = proportion of MCV2 uptake, q = 1-p = 50%, d = a margin of error (5%), Z_1-α/2_ = level of confidence interval, 1.96 (95% CI).

Then, by considering 10% non-response rates, the required final sample size was 424.

There are a total of nineteen kebeles in Shebel Berenta district. Out of the total, eight kebeles were selected using a computer-generated random sampling system. The study participants were mothers who had children aged 24–35 months in the selected kebeles. Then, the sample size was proportionally allocated to each selected kebele. A sampling frame (a list of mothers who had children aged 24–35 months) was prepared at the health post, which was obtained from the family folder (community health information system). Finally, the required subjects were chosen by a simple random sampling technique using a list of children aged 24–35 months.

### Operational definitions

#### Measles second dose vaccine uptake

It is the uptake of the measles second dose vaccine after being vaccinated for measles first dose ^35^.

#### Up-to-date vaccination

The proportion of children aged 24–35 months who had received the measles second dose of vaccine before the age of 24 months^35^.

### Study variables

#### Dependent variables

Measles second dose vaccine utilization.

#### Independent variables

Family size, educational status, marital status, religion, age of the mother, residence, occupation, age of the child, sex of the child, number of births, birth order, distance to health facility, place of delivery, antenatal care service, awareness of measles second dose schedule, knowledge on vaccine preventable disease, utilization of other vaccine antigen such as BCG, MCV1, Pentavalent3, OPV3, Pneumococcal vaccine3, MCV1 and vitamin A supplementation.

### Data collection methods and procedures

A structured, pretested face-to-face interviewer-administered questionnaire adapted from different published literature^17,25–29,36^ and Ethiopia Mini Demographic Health Survey 2019^22^ was used to collect socio-demographic, socioeconomic, and maternal and health facility-related variables, and child vaccination status. Participants were approached and interviewed after explaining the purpose of the study and requesting to participate. Six well-trained public health professionals with previous experience in data collection have participated in the data collection. The data collection process started immediately after preparing the list of children aged 24–35 months at the health post from the selected kebeles. The vaccination information of the children was obtained by requesting mothers or caregivers to show the vaccination cards to the data collectors and mothers' or caregivers' verbal reports if the vaccination cards were not available.

### Data quality assurance

The data collection tool was prepared in English, translated into the local language (Amharic), and then returned to English to ensure consistency. Supervisors and data collectors have received two-day data collection training. The training mainly focused on data quality, confidentiality, and privacy. Sampling procedures and instruction sheets were prepared and given to data collectors and supervisors. One week before the actual fieldwork, a pretest was conducted on 5% of mothers/caregivers with children aged 24–35 months from other kebeles that did not participate in the actual study, and amendments were made based on the results of the pretest. The data were checked for completeness and accuracy by investigators and supervisors daily.

### Data processing and analysis

The data was cleaned manually, coded, and entered into Epi Data 6.4 and exported to SPSS version 25 software for further analysis. Descriptive statistics such as frequency, mean, median and proportion were used to describe the study population concerning relevant variables. Before the analysis was done, the assumptions of the binary logistic regression model were checked. Then, bivariate analysis was carried out to find candidate variables for multivariate analysis. Those variables with a p-value < 0.25 in the bivariate analysis were included in the multivariate analysis to adjust for confounders. An adjusted odd ratio with 95% CIs was estimated to identify factors associated with measles second dose vaccine uptake, and they were declared statistically significant at a p-value < 0.05. Hosmer–Lemeshow’s goodness of fit test model coefficient was found to be insignificant with a large p-value (0.89), which indicates the fitness of the model.

### Ethical consideration

This study involves human subject and all research methods and procedures were performed in accordance with the Declaration of Helsinki and approved by the Debre Markose University Health Science College Institutional Research Ethics Review Committee (IRERC). Further supporting letters were also obtained from the Shebel Berenta district health office. After the purpose and objective of the study had been explained, informed consent was obtained from each subject. Confidentiality of information was maintained, and the collected data was kept in the form of a file in a secure place where no one could access it except the investigators. Mothers/caretakers with unvaccinated or seriously ill children during data collection were advised by data collectors to go to the nearby health post and cluster health center.

### Consent to participate

All procedures involving human subject were approved by Debre Markose University, Health Science College Institutional Research Ethics Review Committee (IRERC). Verbal consent was obtained from all subjects.

## Results

### Socio-demographic characteristics of mothers and children aged 24–35 months

A total of 418 children aged 24–35 months were included in the study, giving a response rate of 98.58%. Of the total participants, 266 (63.6%) of mother-care takers were aged between 20 and 34 years old, with a median age of 31 years. The majority of study participants (95%) were married. About 386 (92.7%) participants were from rural residences, and the majority of respondents (368 (88%) were Orthodox in religion, followed by Muslims, which are 50 (12%). Regarding educational level, 211 (50.5%) respondents did not attend formal education. Concerning occupation, 334 (79.9%) were housewives. Among all respondents, only 122 (29.2%) were exposed to the public media at least once a week. More than half (52.6%) of respondents had 1–3 family sizes (Table 1).

**Table 1:** Socio-demographic characteristics of children aged 24–35 months in Shebel Berenta district, Northwest Ethiopia, 2022.

Variable	Category	Frequency	Percentage % (N = 418)
Residence	Urban	32	7.7
	Rural	386	92.7
Respondents relationship with index child	Mother	359	85.9
	Father	32	7.7
	Grandfather/grand mother	14	3.3
	Aunt	7	1.7
	Siblings > 18 years old	6	1.4
Sex of child	Male	192	45.9
	Female	226	54.1
Mother/caretakers' age	< 20	27	6.5
	20–34	266	63.6
	35–49	111	26.6
	> 49	14	3.3
Mother's/caretakers' educational status	No formal education	211	50.5
	Primary (From Grade 1–8)	155	37.1
	Secondary (From Grade 9 and above)	52	12.4
Marital status of the mothers/caretakers	Married	397	95
	Single	2	0.5
	Divorced	11	2.6
	Widowed	4	1
	Separated	4	1
The mothers'/caretakers' religion	Orthodox	368	88
	Muslim	50	12
Father's educational status	No formal education	224	53.6
	Primary (from grade 1–8)	143	34.2
	Secondary (from grade 9 and above)	51	12.2
Occupation of the mothers/caretakers	Housewife	334	79.9
	Merchant	43	10.3
	Government Employee	9	2.2
	Farmer	19	4.5
	Day Laborer	13	3.1
Household family size	1–3	220	52.6
	4–5	142	34
	≥ 6	56	13.4
Child birth order		78	18.6
	> 1	340	81.4
Alive mothers	Yes	407	97.3
	No	11	2.6
Public media exposure of mother/caretaker	Not at all	165	39.5
	Less than once a week	131	31.3
	At least once a week	122	29.2

### Children’s utilization of other vaccine antigens and Vitamin A supplementation

The majority of children (77.8%) had vaccination cards, while the rest of the participants’ data were gathered through mothers’ or carers' recall. Around 324 (77.5%) and 296 (70.8%) of participants received Bacillus Calmette-Guerin (BCG) and measles-containing first dose (MCV1) vaccinations, respectively. Of the total children, 345 (82.5%) were given pentavalent and pneumococcal conjugate antigens. Regarding vitamin A supplementation, 225 (53.8%) children received vitamin A at the age of six months, while 235 (56.2%) received vitamin A in two doses or more within the age of 24 months (Table 2).

**Table 2:** Children’s utilization of other vaccines in Shebel Berenta district, Northwest Ethiopia, 2022.

Variable	Category	Frequency	Percentage % (N = 418)
Age of children in months	24–29	207	49.5
	30–35	211	51.5
Vaccination card availability	Yes	325	77.8
	No	93	22.2
Baccilus Calmette-Guerin /BCG/	Vaccinated	324	77.5
	Not Vaccinated	94	22.5
BCG scar	Yes	291	89.8
	No	33	10.2
Measles first dose vaccine /MCV1/	Vaccinated	296	70.8
	Not Vaccinated	122	29.2
Pentavalent Vaccine 3	Vaccinated	345	82.5
	Not Vaccinated	73	17.5
Pneumococcal Conjugate Vaccine 3	Vaccinated	347	83
	Not Vaccinated	71	17
Oral Polio Vaccine 3	Vaccinated	345	82.5
	Not Vaccinated	73	17.5
Vitamin A at 6 month (one dose)	Received	225	53.8
	Not Received	193	46.2
Vitamin A two dose and above	Received	235	56.2
	Not Received	237	43.8

### Magnitude of measles second dose vaccine uptake (MCV2)

Overall, the uptake of the measles second dose vaccine (MCV2) among children aged 24–35 months in the Shebel Berenta district, Northwest Ethiopia, was 41.39%. The top reason for not being vaccinated for the measles second dose vaccine was that mothers or caretakers were unaware of the need to return for a second dose of the measles vaccine, 154 (62.60%). Non-compliance with the vaccination schedule was the second reason attributed to 19.92% of children being unvaccinated for the measles-containing second dose vaccine. In addition, fear of adverse effects and overlapping with working time were other reasons that accounted for 6.91% and 4.07% of unvaccinated children, respectively. Furthermore, lack of faith in immunization and overcrowding of vaccination centers were attributed to 4.07% and 2.44% of children being unvaccinated for MCV2, respectively.

### Maternal/caretakers related characteristics

About, 167 (40.0%) of respondents know only one type of vaccine preventable disease and 333 (79.7%) have ever gone for vaccination service. Approximately half of the participants heard about the measles vaccination of children, and 170 (40.7%) respondents were aware of MCV2 vaccination schedule. Participants’ main source of measles second dose vaccine awareness was health workers (91.23%). Other sources for MCV2 awareness were local leaders (5.2%), religious leaders (0.58%), neighbors (0.58%), radio (0.58%), television (0.50%), and others (1.27%). Among respondents, 105 (25.1%) were returned without vaccinating the child after going to the vaccination center mainly due to unavailability of vaccinators during the vaccination session. The second top reason for mothers or caregivers returned without vaccinating their children was child being sick (17.14%). Vaccine unavailability (20%) and long waiting time at vaccination center (20%) were also other contributing reasons for mothers or caregivers returned without vaccinating their children.

### Respondents’ access to health care service related characteristics

About 240 (57.4%), and 235 (56.2%) the participants attended antenatal care and gave birth at health institutions respectively. In the same way, only 141 (33.7%) respondents attended postnatal care for the index child. The majority (60.5%) of respondents walk to the vaccination center at a distance of less than 30 min (Table 3).

**Table 3:** Maternal/caretakers access to health care service in Shebel Berenta district, Northwest Ethiopia, 2022.

Variable	Category	Frequency	Percentage % (N = 418)
Antenatal care visits at health facility during last pregnancy	Yes	240	57.4
	No	178	42.6
Place of delivery for index child	Home	183	43.8
	Health institution	235	56.2
Postnatal care at health facility for index child?	Yes	146	34.9
	No	272	65.1
Average distance to vaccination center in minutes	≤ 30 Minutes	264	63.2
	> 30 Minutes	154	36.8

### Factors associated with Measles second dose of vaccine uptake

In bivariate analysis, variables such as respondent's relationship with index child; mother/caretakers' age, mother's/caretakers' education status, father's education status, household family size, child birth order, media exposure, child vaccination status for the other scheduled vaccine, awareness about MCV2, antenatal care in last pregnancy, place of delivery, postnatal care, and distance to vaccination center were significantly associated with measles second dose vaccine uptake of children aged 24–35 months, and considered them for further analysis.

In multivariate logistic regression analysis, mothers'/caregivers' awareness of measles second dose vaccine, postnatal care visit, child vaccination status to the other scheduled vaccine and distance from vaccine center were remained statistically significantly associated with measles second dose vaccine uptake. As a result, the odds of children measles second dose vaccine uptake was 8.92 times higher among mothers/caretakers who aware about the measles second dose vaccine compared to mothers/caretakers who didn’t aware about measles second dose vaccine (AOR: 8.92, 95% CI 5.27, 15.1). Similarly, children who were vaccinated to other scheduled vaccines were 3.88 times more likely to take measles second dose vaccine compared to children who had not vaccinated to other scheduled vaccines (AOR: 3.88, 95% CI 2.23, 6.73).

The odds of measles second dose uptake among mothers who were attend postnatal care service at health institution for the index child had 4.78 times more likely to vaccinate their child for measles second dose than their counter parts (AOR: 4.78, 95% CI 1.5, 15). This study also found that distance from the vaccination center is related to children's uptake of the measles second dose of vaccination. Mothers/caretakers walked greater than 30 min to reach the nearest vaccination center had less likely to vaccinate their children to measles second dose vaccine by 79% compared to children whose mothers/caretakers walked less than 30 min. (AOR: 0.21, 95% CI (0.06, 0.77) (Table 4).

**Table 4:** Multivariable logistic analysis of factors associated with MCV2 uptake among children aged 24–35 months in Shebel Berenta district, Northwest Ethiopia, 2022.

Variables		MCV2		OR with 95% CI		P-value
		Yes	No	COR	AOR	
Maternal/caretaker postnatal care service	Yes	111	35	10.74 (6.68, 17.25)	4.78 (1.5, 15.34)	0.008
	No	62	210	1	1	
Mothers/caretakers awareness about Measles second dose of vaccine and its schedule	Yes	163	8	10.79 (7.19, 16.19)	8.92 (5.27, 15.1)	0.00
	No	173	173	1	1	
Average distance to vaccination center	≤ 30 min	143	121	1	1	
	> 30 min	30	124	0 .27 (0.13, .34)	0.21 (0.06, 0.77)	0.019
Other scheduled vaccines	Received	94	13	4.05 (3.17, 5.16)	3.88 (2.23, 6.73)	0.00
	Not received	173	245	1	1	

AOR: Adjusted odd ratio, COR: Crude odd ratio, 1: reference group.

## Discussion

In this study, the uptake of the measles second dose vaccine among children aged 24–35 months was 41.39%, which was similar to studies conducted in Indonesia (54%)^37^, Sub-Saharan Africa (44.77%)^25^, the urban area of North Shoa Zone, Central Ethiopia (42.5%)^28^ and the WUENIC 2022 report in the Africa region (45%), and Uganda (46.38%)^38^. However, it is lower than the studies done in China (68.2%)^39^, Trans Nzoia County, Kenya (56.2%)^40^, and Shendi & Almatama Localities, River Nile State, Sudan (84.8%)^41^. The result is still low compared with 2020 WHO report in which the global coverage for measles with the second dose was 70%, western Pacific 94%, eastern Mediterranean 76%, Europe 91%, South East Asia 78%, and America 73%^42^. Similarly, the finding of this study was also lower than the WUENIC 2022 reports in South Africa (86.61%), Rwanda (91%), Eritrea (77.78%), Sudan (63.02%), and Ethiopia (48%)^38^. Despite the study finding, the expected coverage for measles second dose is ≥ 95%^15,43^. A possible explanation could be a lack of awareness among mothers and caregivers about the measles second dose vaccine schedule, difference in health service accessibility and socio-demographic characteristics, and the attitude of populations toward the value of measles immunization^25,44,45^. Interruption of other services provided to the children could also an extra probable reason for the observed variation. Similarly, lack of knowledge regarding the benefits of vaccination in mothers/caregivers and community leaders is one of the greatest root causes of low coverage and under vaccination^46^. On the other hand, this finding is higher than the studies conducted in Ghana (23.9%)^47^, Kenya (17.9%)^26^, the Ethiopian demographic health survey (9%)^22^, and WUENIC 2022 reports in Nigeria (21.17%) and Somalia (7.75%)^38^. This variation might be due data collection methods, difference in introduction of the measles second dose as routine immunization program and environmental factors between the studies.

Out of 424 tudy participants, only 41.39% of them had received MCV2. The major reason for not vaccinating MCV2 was unaware of the need to return for MCV2 (62.6%) and non-compliance with the vaccination schedule (19.92%). This may be because MCV2 was recently introduced as a routine immunization program in Ethiopia. The other possible reason for not taking MCV2 is political crises that may disturb the health service system and make it difficult for vaccinators to travel in the communities. Furthermore, the COVID-19 pandemic may divert the focus of the health system away from the provision of the measles vaccination program. Similarly, 25.1% of mothers returned without vaccinating the child after going to the vaccination center mainly due to the unavailability of vaccinators during the vaccination session. The possible reason for this event could be that most of the health facilities were predominately health centers with a limited number of staff, which led to staff over workload. Our study also found that nearly sixty percent of participants were unaware of the MCV2 vaccination and its schedule. The possible reason for this low level of awareness might be due to a lack of awareness creation in the community during the launch of MCV2 in the National Immunization. Besides, poor communication between healthcare staff and the community about the appropriate ages for vaccination may contribute to a low level of awareness. This low level of awareness among participants may also be an indication that health professionals have not been involved in awareness creation other than service delivery.

Our study revealed that mothers' or caretakers' awareness of measles second dose of vaccine had a significant association with uptake of measles containing second dose of vaccine. Mothers/caretakers who already aware about the measles second dose vaccine and its schedule were almost 9 times more likely to vaccinate their children for measles second dose vaccine to compared to mothers/caretakers who didn’t know about measles containing second dose. This study is in line with two studies done in Kenya^26,27^ and another studies carried out in other part of Ethiopia^28^. The possible reason might be due to the fact that mothers/caretakers who are well informed about the need for measles containing second dose of vaccination and the age at which the child should vaccinated for measles containing second dose vaccine leads to returning to the immunization center for vaccination service^48^. Failure to provide adequate information to the mothers/caregivers by the health workers may result in a reduction in the seeking of health services.

Regarding children vaccination status of other scheduled vaccine, children who vaccinated to other vaccines were almost 4 times more likely to be vaccinated for measles second dose than children who did not vaccinated. This finding is similar to a study done in Kenya^26^ and Ethiopia^29^. A possible explanation is that mothers/caretakers who vaccinate their children to other vaccines may have received health education during their children’s earlier vaccinations. To improve efficiency and effectiveness of vaccination system, it is practical to consider if the administration of MCV2 can be combined with other vaccinations or child health interventions. This can be very attractive to parents as it reduces the number of visits they need to make^35^.

In this study, mothers with postnatal care visit at health institution had more 4 times more likely to vaccinate their children for measles second dose vaccine than their counterparts. This is consistent with studies done in Democratic Republic of Congo^49^, Armenia^50^ and Sub-Saharan Africa^25^. In fact, attending postnatal care visit is a health seeking behavior of women/caregivers. So when women seek to see a health care professional, they might be more likely to seek care for their child as well, and have the opportunities to communicate with health care providers to hear about the benefit of second dose measles vaccination and the importance of completing it^50^. In addition, when mothers/caretakers visit to health facilities, they may informed about the schedule for MCV2 vaccination by health professional^25^. Furthermore, mothers and caretakers who had PNC visits may get full information related to maternal and child care, as well as the importance of vaccine-preventable diseases. Overall, the impact of postnatal care information mainly operates through the role of clinicians in the process of providing the right and up-to-date information to mothers and caretakers.

The distance from the vaccination center affects children's uptake of measles second dose vaccination. Mothers/caretakers walked greater than 30 min to reach the nearest vaccination center had less likely to uptake of measles second dose vaccine compared to children whose mothers/caretakers walked less than 30 min. The result is in line with studies done in Kenya^27^, Shenzhen, China^51^, Sub-Saharan Africa^25^ and Ethiopia^28,52,53^. The possible reasons could be poor accessibility of immunization centers due to sparse population distribution, lack of suitable roads and transport access, travel phobia, motion sickness, and travel costs to reach health facilities. In addition, this might be due to the mother’s given time commitment and workload from household duties, and mothers or caretakers who traveled a great distance might choose not to bring their children to the vaccination site.

Since the study used a quantitative study approach, it may have missed factors that may have been triangulated using a qualitative approach. Moreover, this study did not show the causal-effect relationship.

## Conclusion

The current study showed that measles second dose vaccine uptake in the current study area was too low compared to the WHO target (> 95%). The study also found significant variables that affect measles second dose vaccine uptake of children aged 24–35 months, such as maternal/caregiver’s awareness on measles second dose vaccine and schedule, PNC visit, chld vaccination status of other scheduled vaccine and distance from health/vaccination center. Therefore, interventions that strengthen the health-seeking behavior of mothers/caregivers and the arrangement of vaccination center in nearby residences should be warranted. Targeted communication and women's conferences to emphasise the importance of MCV2 vaccination and schedule adherence should be strengthened. In addition, the district health office should use micro-planning tools such as social mapping to identify hard-to-reach areas or villages; teach community leaders to mobilise their families to get their children vaccinated; monitor their community to identify children under one year of age and those unreached, provide supportive supervision for health extension workers, and arrange functioning vehicles with regular access to fuel and maintenance for vaccinators. Furthermore, regional, zonal, and district health bureaus should strongly sensitize the need for measles-containing second-dose vaccine uptake through radio, television, and other methods.

## Data Availability

All major data have been analyzed and presented in the manuscript.

## Abbreviations

AOR: Adjusted Odds Ratio
BCG: Bacilli-Calmette-Guerine
CHAI: Clinton Health Access Initiative
CHIS: Community health information system
COVID-19: Corona Virus Disease 2019
COR: Crude odds ratio
CI: Confidence interval
ECSA: Ethiopian central Statistics agency
EDHS: Ethiopian Demographic and Health Survey
EPI: Expanded Program of Immunization
HMIS: Health Management Information System
GAVI: Global Alliance for Vaccine and immunization
IA2030: Immunization Agenda 2030
IgM: Immunoglobulin M
MCV1: Measles containing vaccine first dose
MCV2: Measles containing vaccine second dose
OPV3: Oral Polio vaccine third dose
SDG: Sustainable Development Goals
SPSS: Statistical Package for Social Science
UNICEF: United Nations international Children and Education Fund
WHO: World Health Organization
